# Recent advances in and applications of ex vivo drug sensitivity analysis for blood cancers

**DOI:** 10.1007/s44313-024-00032-8

**Published:** 2024-11-06

**Authors:** Haeryung Lee, Nahee Ko, Sujin Namgoong, Seunghyok Ham, Jamin Koo

**Affiliations:** 1 Department of Chemical Engineering, Hongik University, Seoul, 04066 Republic of Korea; 2 ImpriMedKorea, Inc., Seoul, 03920 Republic of Korea

**Keywords:** Drug sensitivity, Blood cancers, Precision medicine, Machine learning, Prognosis

## Abstract

Blood cancers, including leukemia, multiple myeloma, and lymphoma, pose significant challenges owing to their heterogeneous nature and the limitations of traditional treatments. Precision medicine has emerged as a transformative approach that offers tailored therapeutic strategies based on individual patient profiles. Ex vivo drug sensitivity analysis is central to this advancement, which enables testing of patient-derived cancer cells against a panel of therapeutic agents to predict clinical responses. This review provides a comprehensive overview of the latest advancements in ex vivo drug sensitivity analyses and their application in blood cancers. We discuss the development of more comprehensive drug response metrics and the evaluation of drug combinations to identify synergistic interactions. Additionally, we present evaluation of the advanced therapeutics such as antibody–drug conjugates using ex vivo assays. This review describes the critical role of ex vivo drug sensitivity analyses in advancing precision medicine by examining technological innovations and clinical applications. Ultimately, these innovations are paving the way for more effective and individualized treatments, improving patient outcomes, and establishing new standards for the management of blood cancers.

## Introduction

Blood cancers, including leukemia, multiple myeloma (MM), and lymphoma, represent a significant burden on global health and affect several million patients worldwide [1]. Traditional treatment approaches for these malignancies, such as chemotherapy and radiation, often have limitations because of their lack of specificity and associated toxicities. In recent years, precision medicine has emerged as a transformative approach in oncology, offering the promise of more effective and personalized treatments tailored to individual patient profiles [2–4]. Central to this approach is the use of genotyping analysis to reveal the trait(s) of each patient’s tumor. More recently, the development and use of ex vivo drug sensitivity analysis have been reported, which allows the testing of patient-derived cancer cells against a panel of therapeutic agents to predict clinical responses [5–10]. This technology not only aids in identifying the most effective treatments for individual patients but also helps to understand resistance mechanism(s), thereby paving the way for more targeted and successful therapeutic strategies.

A critical challenge in ex vivo drug sensitivity analysis is to keep patient-derived cells alive and functionally intact outside the human body. Initial applications of this technology in blood cancers, particularly acute myeloid leukemia (AML), have demonstrated promising results with the successful development of media that maintains cells viable for assaying [10–12]. The relative homogeneity of leukemic cells when sampled, compared to the heterogeneous cell populations found in solid tumors, also contributed to the success of the approach. These early studies highlight the potential of ex vivo assays to accurately predict patient responses to various treatments, offering a glimpse into the future of personalized medicine. However, significant variability in assay protocols and results across different institutions underscores the need for standardized methodologies to ensure reproducibility and reliability.

Recent advancements in ex vivo drug sensitivity analysis have focused on refining the metrics used to describe drug response curves, moving beyond simple IC_50_ values to include more comprehensive indices such as the area under the curve (AUC) and drug sensitivity scores (DSS) [13–16]. The integration of machine learning algorithms has further revolutionized the field, enabling the analysis of complex datasets to identify patterns and predict patient responses with a greater accuracy [17–20]. Machine learning models can incorporate a wide range of variables, including genetic and molecular profiles, to enhance the predictive power of ex vivo assays. Additionally, a growing emphasis has been observed on analyzing drug combinations, recognizing that combination therapies often yield superior results compared with single-agent treatments. By systematically evaluating multiple drug combinations ex vivo, researchers can identify synergistic interactions and optimal therapeutic strategies for individual patients, further advancing the precision medicine approach for treating blood cancers [16, 21–23].

**Fig. 1:**
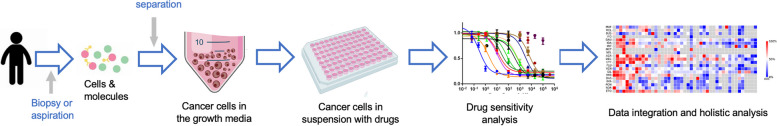
Schematic of ex vivo drug sensitivity analysis using the patient-derived cells

This review aims to provide a comprehensive overview of the latest advances in ex vivo drug sensitivity analysis (Fig. 1) and its applications in precision medicine for blood cancers. We will explore technological innovations that have enhanced the predictive accuracy and clinical utility of ex vivo assays, with a particular focus on leukemia, MM, and lymphoma. In addition, we discuss the integration of mathematical modeling methods and the evaluation of drug combinations, highlighting how these approaches improve prediction of treatment outcomes. By examining both the current state and future potential of ex vivo drug sensitivity analysis, this review describes its critical role in advancing personalized oncology. Ultimately, we aim to illustrate how these innovations are paving the way for more effective and tailored therapies for blood cancer patients.

### Advances in drug sensitivity metrics

Ex vivo drug sensitivity analysis was performed by incubating patient-derived cells with the drug(s) of interest and measuring changes in growth and/or viability with respect to various drug concentrations. The resultant data are often called dose–response curves and are typically fitted using logistic regression or Hill’s equation [13, 24]. From the latter, a parametric value known as the IC_50_ can be estimated, which has traditionally been used to represent sensitivity to a given drug. The parameter represents the concentration at which the growth or viability is inhibited or reduced by half. The IC_50_ values of drugs against cell lines have commonly been reported in the literature, including those of anti-cancer drugs against cell lines mimicking blood cancers, such as HL-60 and DS [25–27].

The popularity of IC_50_ stems from its simplicity and ease of derivation from dose–response curves, which is a standard method in pharmacological studies [13, 24, 28]. However, it also has limitations, primarily because of its inability to fully represent the characteristics of the respective drug sensitivity analysis results, including the slope and maximal effect. Attempts have been made to develop and apply other metrics that capture characteristics not fully described by the IC_50_ values (Table 1). The AUC and E_max_ were proposed, followed by the DSS [13, 14]. These metrics provide a more holistic view by considering the entire dose–response relationship and integrating multiple data points for computation. The differential drug sensitivity score (dDSS) is the latest metric that enhances DSS by identifying cancer-selective drugs, thereby aiding the personalization of cancer treatment [14, 34]. dDSS considers not only the efficacy of a drug across a range of concentrations but also its differential effects on cancer versus non-cancerous cells, allowing for more precise therapeutic targeting​.

**Table 1:** Metrics used to describe drug sensitivity analysis results

Metric	Description	Applied disease(s) and reference(s)
IC_50_	The concentration of the drug by which growth or viability is inhibited or reduced by half: $y=y_{LL}+\frac{y_{UL}-y_{LL}}{1+{(\frac C{{IC}_{50}})}^m}$	ALL [5], MM [27], NHL [26]
E_max_	The (average) remaining viability of cells at maximal concentration(s): ${E}_{max}=\sum_{i=1}^{n}{y}_{{C}_{i}}$	AML [28, 29], NHL [30]
AUC	The area under the dose–response curve, usually obtained at a fixed time point like in below: $AUC={\int }_{{C}_{i}}^{{C}_{f}}y dC$	AML [29, 31], MM [32], NHL [33]
DSS	The integral of response over the dose range that exceeds a given minimum activity level (*C*_*min*_): $DSS= {\int }_{{C}_{min}}^{{C}_{f}}(100\%-y) dC$	AML [14], ALL [34], MM [6]
dDSS	The difference between DSS quantified in patient cells (patient DSS) and the average drug response of control samples: $dDSS= {\int }_{{C}_{min}}^{{C}_{f}}(z-y) dC$	AML [14], ALL [34]

^a^Parameters: *y*, viability of cells (%); *y*_*UL*_, upper limit of y; *y*_*LL*_, lower limit of y; *C*, concentration of the drug (nM or$\mu$M); *m*, slope of the dose–response curve at IC_50_; *C*_*i*_, the lowest concentration of interest; *C*_*f*_, the highest concentration of interest; *z*, viability of controls (%)

^b^*Abbreviations*: *ALL* Acute lymphoblastic leukemia, *MM* Multiple myeloma, *NHL* Non-Hodgkin lymphoma

Recent studies have illustrated how drug sensitivity metrics can be used to predict treatment outcomes in blood cancer patients treated with their respective drug(s). For example, Park et al. showed that the E_max_ of venetoclax can be used to predict early death in AML patients treated with venetoclax plus a hypomethylating agent [29]. In contrast, AUCs measured during ex vivo sensitivity analyses are reportedly associated with biomarkers of poor prognosis and later relapse in patients with MM [32]. Yadav et al. suggested that dDSS can be used to compare the efficacy of anti-cancer drugs for a given AML patient based on the pioneering study involving 16 patients [14]. The ability to compare drugs based on sensitivity analysis is crucial for identifying the most effective drug(s) for a given patient. These promising results invite further research to understand the applicability of this metric in large cohorts.

### Analysis of sensitivities to drug combinations

The use of drug combinations in cancer treatment is crucial for enhancing drug sensitivity and maximizing therapeutic outcomes because they can leverage multiple therapeutic mechanisms to target various pathways within cancer cells. This strategy is particularly effective in overcoming drug resistance, reducing treatment failures, and improving overall efficiency compared with single-agent therapies. As such, most therapies that blood cancer patients receive to date are based on drug combinations, such as venetoclax plus a hypomethylating agent for AML [35–37], rituximab plus vincristine plus cyclophosphamide plus doxorubicin plus prednisone for diffuse large B-cell lymphoma [38], or bortezomib plus lenalidomide plus dexamethasone for MM [39, 40]. These examples reflect the emerging need to evaluate the effectiveness of drug combinations in ex vivo sensitivity assays, particularly if the results are to be used to support personalized treatment.

Over the past decade, multiple pioneering attempts have been made to analyze patient-derived cell sensitivity to drug combinations (Table 2). The pioneering method in this area is the Chou-Talalay method, which evaluates the additivity, synergism, or antagonism of drug combinations using combination indices [21]. The latest methods have attempted to overcome the limitations of the Chou-Talaly method by adopting more quantitative and/or advanced models or by enabling an exhaustive search of two or more drug combinations. For example, Synergy Finder Plus utilizes a greater variety of models, such as Loewe additivity (LOEWE) and Bliss independence (BLISS), to provide a more comprehensive analysis of drug combinations [41]. Chen et al. employed artificial intelligence (AI) methods to train models that predict synergism between drugs based on datasets of 583 different drug combinations tested against 39 human cancer cell lines [45]. More recently, researchers tested combinations of three drugs against patient-derived cells and identified the most effective combination based on the overall response within a composite design space [22]. The authors used these results to optimize drug combinations for relapsed/refractory non-Hodgkin lymphoma (NHL) and reported an enhanced response rate and survival. Future clinical studies employing these novel methods to identify optimal drug combinations may provide evidence to revolutionize cancer treatments.

**Table 2:** The methods reported in the literature for analyzing drug combinations via sensitivity assays

Name and reference	Description	Applied disease(s) and reference(s)
Synergy finder plus [41]	Software for analyzing drug combination synergy and sensitivity using models such as HSA, BLISS, LOEWE, and ZIP	Various cancer types [41], COVID-19 [42]
Cross-design for drug combination sensitivity score and synergy analysis [43]	Evaluation of drug combination sensitivity and synergy by fixing one drug at a constant concentration and varying the doses of another	Various cancer types [43], AML [44]
Multi-task learning-based synergy prediction [45]	A multi-task learning and deep neural networks-based method to predict drug combination synergy and monotherapy sensitivity	Various cancer types [45]
Quadratic phenotypic optimization platform (QPOP) [46]	Optimizing drug combinations by analyzing drug responses using an orthogonal array composite design to efficiently test multiple drugs across various concentrations	NHL [46], various cancer types [47–49]

### Analysis of sensitivities to advanced therapeutics

Antibody-based therapies for blood cancers, including AML, NHL, and MM have emerged as the cornerstones for the treatment. These therapies leverage the specificity of antibodies against the target cancer cells, thereby offering a promising avenue for precision medicine. Recently, antibody–drug conjugates (ADCs) and cell therapies employing engineered T cells have been developed and shown promising results against various diseases, including blood cancers. These advanced therapeutics (Table 3) cure patients based on complex mechanisms (s) of action (MoAs), wherein non-cancerous cells, especially immune cells, contribute to the elimination of tumors. Therefore, drug sensitivity assays must be modified to incorporate additional reagents and/or moieties that participate in MoAs.

**Table 3:** Antibodies, antigens, and ex vivo drug sensitivity assays used to test the utility and applicability of the antibodies against the targeted diseases

Antibody(s)	Antigen(s)	Method used to analyze drug sensitivity	Applied disease(s) and reference(s)
Gemtuzumab	CD33	Colony formation (ex vivo), cytotoxicity (in vitro)	AML [49]
AMG 330	CD33, CD3	Cytotoxicity (ex vivo, in vitro)	AML [50]
IMGN632	CD123	Colony formation (ex vivo), cytotoxicity (in vitro), internalization/processing (in vitro)	AML [51]
JNJ-67571244	CD33, CD3	Cytotoxicity (ex vivo, in vitro)	AML [52]
NKp46-CD16a-NK Cell Engager	CD123	Cytotoxicity (ex vivo, in vitro), NK Cell activation (in vitro)	AML [53]
Obinutuzumab, Rituximab	CD20	Cytotoxicity (in vitro), IFNγ Release (in vitro), B-cell depletion (ex vivo)	NHL [54, 55]
STRO-001	CD74	Cytotoxicity (in vitro)	NHL [56]
Daratumumab	CD38	Cytotoxicity (ex vivo, in vitro), NK Cell expansion (ex vivo) & activation (in vitro)	MM [57–59]
Elotuzumab	SLAMF7	Cytotoxicity (ex vivo), enzyme-linked Immunosorbent assay (ex vivo)	MM [60]

*Abbreviations*: *AML* Acute myeloid leukemia, *NHL* Non-Hodgkin lymphoma, *MM* Multiple myeloma, *NK* Natural killer, *IFN* Interferon

Drug sensitivity analyses have been instrumental in the evaluation and refinement of antibody-based therapies for AML. One notable example is gemtuzumab ozogamicin, an anti-CD33 ADC. Early drug development research highlighted its potent and selective cytotoxic effects on CD33 + AML cells both in vitro and in vivo [49]. This drug demonstrated significant activity in preclinical models and early-phase clinical trials, ultimately leading to its approval by the Food and Drug Administration as the first antibody-targeted chemotherapeutic agent for AML. Ex vivo studies demonstrated that the addition of granulocyte colony-stimulating factors enhances the cytotoxicity of the drug against AML cells, providing a rationale for its clinical use [61]. Further studies comparing the drug with novel therapies, such as the TRAIL fusion protein, revealed superior selectivity and activity [62]. Another promising development is the use of bispecific T-cell engagers, such as AMG 330, which target CD33 and CD3 to engage T-cells in AML therapy. Ex vivo drug sensitivity analysis showed the potent activity of AMG 330 against AML cells, supporting its progression into clinical trials [50].

Drug sensitivity analyses have facilitated the development of antibody-based therapies for NHL. Obinutuzumab, a next-generation anti-CD20 antibody, has been tested in vitro and has been shown to induce greater cytotoxicity compared to older antibodies such as rituximab, particularly when combined with ibrutinib [54]. These results were corroborated by in vivo studies highlighting the potential for improved patient outcomes [55]. Ex vivo drug sensitivity analyses are also pivotal for optimizing combination therapies involving antibodies and cytotoxic drugs. Studies have demonstrated that rituximab enhances the efficacy of cytotoxic drugs in neoplastic lymphocytes, paving the way for combination regimens in clinical practice [63]. New developments in this field include targeting CD74 in B-cell NHL with ADC STRO-001, which has demonstrated promising results in recent studies [56].

In MM, drug sensitivity analyses have been instrumental in assessing the impact of novel antibody therapies, such as daratumumab, on natural killer (NK) cells. Daratumumab targets CD38 on myeloma cells and induces NK cell fratricide, leading to NK cell depletion. Ex vivo-expanded autologous NK cells have the potential to overcome this depletion, thereby enhancing the therapeutic efficacy of Daratumumab [57–59]. Moreover, the effects of the drug on NK cells are particularly significant in patients with relapsed or refractory MM patients, which has implications for clinical outcomes [59]. Drug sensitivity analyses have shown that elotuzumab enhances NK cell-mediated cytotoxicity against myeloma cells by upregulating NK cell-enhancing genes, thereby supporting the development of combination therapies for MM. The ex vivo sensitivity profiles of various treatments, including proteasome inhibitors and immunomodulatory drugs, have also been explored, providing insights into the development of drug resistance and the selection of subsequent therapies [60]. Kropivsek et al. demonstrated the clinical utility of sensitivity profiles for treatment optimization in 70 patients with MM [9].

## Conclusion

Ex vivo drug sensitivity analysis represents a significant advancement in precision medicine for blood cancers, particularly AML, MM, and lymphoma. Researchers have greatly enhanced the predictive power and clinical utility of these analyses by refining the metrics used to describe drug response curves and integrating machine learning techniques. The ability to evaluate drug combinations has opened new avenues for identifying synergistic regimens, which are crucial for overcoming drug resistance and improving patient outcomes. Moreover, the ability to assess advanced therapeutics, such as ADCs, underscores their potential for tailoring treatments based on complex MoAs involving the immune system. As the field continues to evolve, the ongoing development and application of ex vivo drug sensitivity analysis are poised to revolutionize precision medicine, ultimately leading to more effective and individualized treatments for patients with blood cancers and other diseases.

## Data Availability

No datasets were generated or analysed during the current study.
